# Are There Neural Overlaps of Reactivity to Illegal Drugs, Tobacco, and Alcohol Cues? With Evidence From ALE and CMA

**DOI:** 10.3389/fpsyt.2022.779239

**Published:** 2022-04-06

**Authors:** HuiLing Li, Dong Zhao, YuQing Liu, JingWen Xv, HanZhi Huang, Yutong Jin, Yiying Lu, YuanYuan Qi, Qiang Zhou

**Affiliations:** ^1^Department of Psychology, Wenzhou Medical University, Wenzhou, China; ^2^The Affiliated Kangning Hospital, Wenzhou Medical University, Wenzhou, China; ^3^Zhejiang Moganshan Female Drug Detoxification Center, Huzhou, China; ^4^Mental Health Education and Counseling Center, Lingnan Normal University, Zhanjiang, China

**Keywords:** neuroimaging, cue-reactivity, tobacco, alcohol, drug

## Abstract

Abuses of most illegal drugs, including methamphetamine, marijuana, cocaine, heroin, and polydrug, are usually in conjunction with alcohol and tobacco. There are similarities and associations between the behavior, gene, and neurophysiology of such abusers, but the neural overlaps of their cue-reactivity and the correlation of neural overlap with drug craving still needs to be further explored. In this study, an Activation Likelihood Estimation (ALE) was performed on brain activation under legal (tobacco, alcohol) and illegal drug cues, for identifying the similarities in brain functions between different craving states. A Comprehensive meta-analysis (CMA) on the correlation coefficient between brain activation and craving scores in the selected literatures with subjective craving reports explained the degree of the craving via brain imaging results. In ALE, co-activation areas of the three cue-reactivity (posterior cingulate, caudate, and thalamus) suggest that the three cue-reactivity may all arouse drug-use identity which is a predictor of relapse and generation of conditioned reflexes under reward memory, thus leading to illegal drug relapses. In CMA, the brain activation was significantly correlated with subjective craving, with a correlation coefficient of 0.222. The neural overlap of tobacco, alcohol and most of the prevalent illegal drug cues not only further helps us understand the neural mechanism of substance co-abuse and relapse, but also provides implications to detoxification. Furthermore, the correlation between brain activation and craving is low, suggesting the accuracy of craving-based quantitative evaluation by neuroimaging remains unclear.

## Introduction

Substance abuse is a major culprit damaging human physical and mental health and can even lead to death. Tobacco, alcohol, and illegal drug abuse are particularly serious. Alcohol and tobacco use cause the loss of more than 250 million disability-adjusted life years to humans, and illegal drugs cost tens of millions (1). Alcohol and tobacco are the most commonly abused legal drugs, but the legalization of common drugs of abuse is arbitrary and there is a lack of scientific and systematic criteria for classifying drugs of abuse (2). This may lead to misconceptions about the harm of each drug, and people may simply assume that the abuse of legal drugs is less important than the abuse of illegal drugs, which may not be the case. Nutt et al. (3) developed a nine-category matrix of harm to classify drugs based on physiological impairment, drug dependence, and social impact, and found that tobacco and alcohol were more harmful than some Class A drugs (the most harmful class according to the UK Misuse of Drugs Act) and that their co-abuse with illegal drugs exacerbated the damage.

Tobacco and alcohol abuse can cause damage to the human body in multiple ways. Alcohol abuse causes impairment in executive function, memory, emotional function, and is also a major risk factor for traumatic brain injury (4). Nicotine abuse is strongly associated with the occurrence of sleep disorders, depression, schizophrenia, and anxiety disorders (5). The abuse of illegal drugs has even more serious consequences, as it can lead to acute or subacute leukoencephalopathy, as well as vascular complications, including vasoconstriction, vasculitis, and hypertension (6); it can also severely impair prospective memory—the higher the frequency of cocaine use, the stronger the degree of memory deficit (7).

Illegal drug abuse is often accompanied by tobacco and alcohol abuse (8). Research has found evidence of co-abuse of alcohol, tobacco and illegal drugs. Smoking rates among methamphetamine abusers typically exceed 80% (9). 86.4% of cocaine abusers reported co-abusing tobacco, 99.4% co-abusing alcohol and 95.1% co-abusing cannabis. In a dire co-substance abuse situation, it cannot be ignored that both tobacco and alcohol abuse have significant effects on illegal drug abuse, and alcohol abuse serves as a mediating factor between tobacco and illegal drug use (10). Some studies have found that simultaneous abuse of alcohol and psychostimulants can lead to neurophysiological dysfunctions, such as decreased antioxidant enzymes in the brain, disruption of learning and memory processes, inadequate brain perfusion, and neurotransmitter depletion; as well as increased heart rate, blood pressure, myocardial oxygen consumption, cellular stress, and increased risk of different types of cancer (11). Joint abuse of cocaine and nicotine enhances co-induced locomotor activity, as well as the induction and expression of locomotor sensitization, making each other mutually reinforcing abuse (12). Thus, the concurrence of tobacco, alcohol and illegal drug abuse is highly harmful.

Since abusers' cravings for tobacco and alcohol increase their cravings for drugs, it is proposed that smoking and drinking cessation should be performed simultaneously with detoxification (13–18). But institutional issues and individual health care providers often skip providing concomitant treatment to tobacco and alcohol abusers. Despite evidence that co-abuse of alcohol and cocaine produces unique neuroadaptations, their concomitant treatment needs are far from being met (19). Among alcohol abusers, methamphetamine is the most commonly co-abused illegal drug, but there is no effective treatment for this methamphetamine addiction comorbidity (20). Exploring the exact relationship between tobacco and alcohol abuse and illegal drug abuse can shed light on this dilemma.

Strong genetic and neurophysiological correlations among tobacco abuse, alcohol abuse and drug abuse have been identified. Research on the genetics of co-drug abusers could help develop more effective treatment programs (21–23). By measuring genetic variation, people initially found a certain genetic correlation between nicotine and marijuana (24). Drug abuse can lead to drug addiction. The widespread changes in hippocampal gene expression in both cocaine dependents and alcohol dependents may reflect neuronal adaptation common to both addictions (25). In terms of neurophysiological changes, when both illegal and legal drugs are abused, their interactive effects on neurophysiological mechanisms exacerbate the damage. After co-abuse of tobacco, alcohol and illegal drugs, the brain and biological mechanisms of abusers will have abnormal changes. It has been found that alcohol will increase the concentration of different psychostimulants and their active metabolites in the blood (26). When alcohol is used in conjunction with these drugs, the pharmacokinetics of methamphetamine, cocaine, and nicotine may change (11). Drinking alcohol alone did not affect the levels of dopamine and serotonin in the striatum and prefrontal cortex, but injecting methamphetamine after previously consuming alcohol somehow enhances methamphetamine-induced dopamine and serotonin (27). It can be seen that the abuse of tobacco and alcohol will aggravate the neurophysiological damage of illegal drugs. In addition, the three drug abuses have a common neurophysiological mechanism, such as the reward circuit of abnormal dopamine release (28). Are there overlaps between different cue-induced craving state?

Exploring the neurophysiological mechanism of craving can not only provide theoretical guidance for the “regression model of craving,” but also provide enlightenment for considering whether the craving for one drug triggers the intake of another addictive substance while solving concomitant drug use. In previous studies, methods of “induction under cues” or “physical withdrawal” are generally used to induce subjects' craving for psychoactive substances (29, 30). The measurement of brain changes under cue induction in neuroimaging only proves that the neurophysiological mechanisms caused by the two inducing conditions are different but cannot prove the exactly differences in craving. Therefore, the accuracy of neuroimaging to assess drug craving is often illustrated by the correlation coefficient between its results and subjective self-evaluated craving scores (31). However, the degree of correlation between drug craving scores and activated brain regions was different in different studies. Therefore, in this meta-analysis, we need to clarify the degree of correlation between cue induction and craving.

In a word, Tobacco, alcohol, and drugs are often abused jointly. They have a certain mutual predictive relationship and a common biological mechanism (32). Since craving is a major cause of relapse, research on the impact of tobacco and alcohol craving on drug relapse is critical. Presently, the similarities between the brain mechanisms of legal drug (tobacco and alcohol) cravings and illegal drug cravings are unclear. This study employed activation likelihood estimation meta-analysis (ALE meta-analysis) to conclude similarities in activated brain areas in drug-dependent patients under induction by legal drug (tobacco, alcohol) and illegal drug cues. We hypothesize that these three cues induce some co-activated brain regions. In addition, a Comprehensive Meta-Analysis (CMA) was performed for the correlation coefficients between the brain activation levels and self-reported scores of the cravings. The level of activation of co-activated brain regions may to some extent represent the degree of craving. The results of this study are expected to provide enlightenment for the treatment sequence of tobacco, alcohol, and drugs and the effectiveness of neuroimaging measurements of drug cravings.

## Methods

### Literature Search

After determining the issue for investigation, three sets of search keywords were determined (each set separated by “or”): (1) related words for craving induction by cues—craving/cue; (2) words related to drug addiction—addiction/drug use/drug abuse/drug dependence/substance use/substance abuse/substance dependence/alcohol/ heroin/cocaine/opiate/cannabis/marijuana/nicotine/smoke/tobacco/MDMA/polydrug; and (3) words related to brain/imaging—fMRI/functional Magnetic Resonance Imaging/BOLD/blood oxygen level dependent/neuroimaging/PET/Positron Emission Computed Tomography/fNIRS/ functional near-infrared spectroscopy. Data bases including Web of Science, PubMed, PsycINFO, CNKI, and others were searched. The publication time was set from January 1975 to March 2021, and the search contents were three sets of search terms connected by “AND.” Supplemental screening was conducted for the included literature.

### Literature Screening

The downloaded literature was screened according to the inclusion criteria: (1) the coordinates of the enhancement point of the drug cue-neutral cue were reported; (2) it uses the statistics contrasts(drug cue > Neutral cue); (2) it was a whole brain study, not a specific brain area study; (3) the drug craving was induced by the cue; (4) it adopted an in-group design—the brain activation areas of drug-dependent patients under drug and neutral cues were compared; (5) research subjects were substance abusers; (6) fMRI, PET, or fNIRS was used; (7) literature review and meta-analysis were excluded; and (8) subject had no mental illness.

### Implementation of Meta-Analysis

#### ALE Meta-Analysis

**Figure 1:**
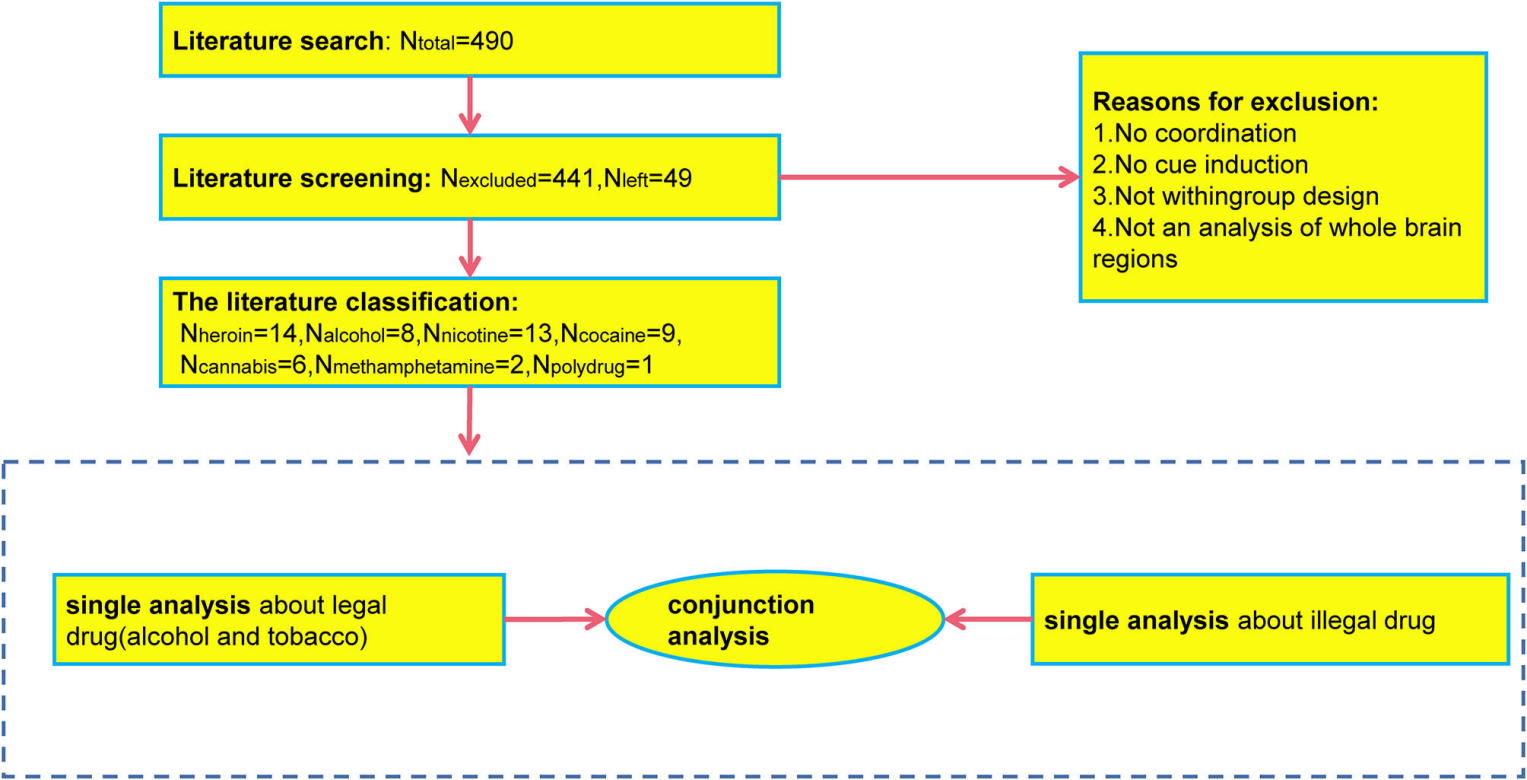
ALE meta-analysis process.

The final coordinates were organized into text, and GingerALE 2.3.6 was used to convert the coordinates based on Talarich template to the coordinates based on Montreal Neurological Institute (MNI) template; to be conservative, according to the recommendations of the ALE instruction manual, the threshold of the diagram of activation likelihood estimation was set to *p* < 0.001 and corrected by the method of Uncorrected P (33). The minimum cluster size was 250 mm^3^ (34), and the default preferences were set. The following meta-analysis was performed: (1) meta-analyses were performed for legal drug-related (tobacco-related and alcohol-related) and illegal drug-related literature separately; (2) a conjunction meta-analysis was performed between legal drug-related (tobacco-related and alcohol-related) literature and illegal drug-related literature, separately (see Figure 1). Each meta-analysis produced their respective activation area pictures and cluster files. Mango4.1 (http://rii.uthscsa.edu/mango/) was used to cover the activation area on the MNI standard brain (http://www.brainmap.org/ale/) (35).

#### Comprehensive Meta-Analysis

##### Effect Size

Of the 49 included articles, two papers reported the correlation coefficient between an activated brain area (drug cue > neutral cue) and craving score; seven papers reported the correlation coefficients between several activated brain areas (drug cue > neutral cue) and craving score. Ultimately, we obtained a total of 26 correlation coefficients as effect sizes.

##### Selection of Models

Current meta-analyses mainly use fixed-effect models or random-effect models. The fixed-effects model assumes that there is only one true effect size behind all studies in the meta-analysis, and that the difference in effect size for each study is due to sampling error. The random effects model assumes that the true effect size is different for each study and that the difference in effect size for each study is due to a combination of the difference in true effect size and sampling error (36). If the total effect sizes from the meta-analysis are not only for the included studies but need to be extended to other groups, we should use a random-effect model (36). Since the age, gender, occupation, etc. of the subjects in the meta-analysis varied, the effect sizes obtained from our meta-analysis could not be limited to just one, so we chose a random-effect model.

##### Test for Publication Bias

Publication bias means that the published research literature does not systematically and comprehensively represent the total body of research that has been done in the field (37). The most effective way to remove publication bias is to increase the sample size (including published and unpublished studies), as a lack of representative sample, particularly of dissertations with insignificant or unpublished findings, may affect the reliability of the meta-analysis results. To address this issue, firstly, we obtained as many unpublished papers as possible during the literature search stage; secondly, in the specific meta-analysis process, we used three methods including funnel plot, Rosenthal's Classic Fail-safe *N*-test, Egger's test to further evaluate publication bias.

##### Comprehensive Meta-Analysis Process

CMA (comprehensive meta-analysis) is a commercial software package dedicated to meta-analysis (www.meta-analysis.com), developed by Borenstein et al. (36). It was released in 2007 with Version 2.0 and above, and is now available in Version 3.0. In our experiments, we used CMA version 2.2. The software has a user-friendly interface, is easy to operate, can import more than 100 kinds of data structures, and can implement advanced statistical analysis functions such as subgroup analysis, meta regression and cumulative meta-analysis.

Using correlation coefficient as the effect size, random effects models were used and CMA 2.2 was adopted for meta-analysis. Methods such as funnel plot, Begg's test, Egger's tests and the Trim and Fill method were used to evaluate the publication bias of this meta-analysis.

## Results

### Description of Included Literature

Of the 49 articles that met the inclusion criteria, one article contained two addiction groups with different lengths of detoxification, one article included two addiction groups with different drug cues and one contained three addiction groups with different addictive substances. There were altogether 53 sub-studies from the above mentioned articles included in this study, and they could be classified by addictive substance, 14 articles explored heroin; 8, alcohol; 13, tobacco; 9, cocaine; 6, marijuana; 2, methamphetamine; and 1, polydrug addiction. With consideration of cue exposure, treatment status of the participants, abstinence of the samples included, and diagnosis modulating the brain reactions to drug cues (38), we collated relevant information from the included literature (See Table 1 in the additional file).

**Table 1:** Characteristics of the study.

**References**	***N***	**Male%**	**Mean age (years)**	**Diagnostic criteria**	**Mean time of drug abuse**	**Daily dose of drug use**	**Withdrawal time**	**Comorbidities**	**Types of cues**	**Imaging technology**	**Questionnaire for cravings**	**Brain regions**	**Correlation coefficient**
**Heroin**													
Wang et al. (39)	32	53	29.19 ± 7.50	DSM-IV	–	–	–		Picture	fMRI			
Hossein Tabatabaei-Jafari et al. (40)	40	100	32.00 ± 4.40		11.35 ± 4.60 years	-	3 months		Picture	fMRI			
Li et al. (41)	18	100	34.60 ± 6.80	DSM-IV	96.30 ± 69.50 months	0.80 ± 0.40 g	6 months		Picture	fMRI			
Chang (42)	10	100	30.70 ± 5.50	DSM-IV	79.30 ± 47.40 months	0.71 ± 0.25 g			Picture	fMRI			
Wang et al. (43)	14	100	41.00 ± 5.60	DSM-IV	58.14 ± 12.27 months	1.07 ± 0.54 g			Picture	fMRI			
Lou et al. (44)	37	100	32.38 ± 1.40	DSM-IV	7.62 ± 1.05 years	0.70 ± 0.15 g			Picture	fMRI			
Wang et al. (45) (short-term withdrawal group)	17	100	33.20 ± 1.40		7.00 ± 1.00 years	0.60 ± 0.10 g	1.2 ± 0.1 months		Picture	fMRI			
Wang et al. (45) (long-term withdrawal group)	17	100	31.80 ± 1.40		8.40 ± 1.10 years	0.70 ± 0.10 g	13.7 ± 0.4 months		Picture	fMRI			
Song et al. (46)	10	100	37.79 ± 6.46	DSM-III R	58.14 ± 12.27 months	1.07 ± 0.54 g	–		Drug	fMRI			
Yang (47)	12	100	33.20 ± 4.31	DSM-IV	10.00 ± 1.30 years	0.25 ± 0.11 g	≤ 1 month		Picture	fMRI			
Zijlstra et al. (48)	40	100	44.50 ± 3.90	DSM-IV	16.00 ± 6.80 years	–	8.1 ± 6.1 weeks		Picture	fMRI			
Shao et al. (49)^*^	30	67	31.00 ± 8.00	DSM-IV	6.00 ± 3.00 years	1.20 ± 0.80 g	9 ± 2 months		Picture	fMRI	11-point Likert scales	Left inferior frontal gyrus	0.554
												Left middle frontal gyrus	0.512
												Left anterior cingulate	0.587
												Right orbitofrontal cortex	0.528
												Right amygdala	0.515
												Right insula	0.509
												Left medial frontal gyrus	0.501
Xiao et al. (50)	14	100	33.2		7.10 years	–	0		Picture	fMRI			
Sun et al. (51)	30	67	30.9	DSM-IV	5.92 ± 3.24 years	1.20 ± 0.80 g	1.90 ± 2.30 months		Video	fMRI			
Totals or samplesize-weighted averages	321	89	34.41 ± 3.81		85.13 ± 30.86 months	0.56 ± 0.23 g							
**Cocaine**													
Zhang et al. (52)	23	74	42.20 ± 7.60	DSM-IV	16.00 ± 9.70 years	1.10 ± 0.70 mg			Picture	fMRI			
Ma et al. (53)	15	100	39.10 ± 8.00	DSM-IV	–	–	14.6 ± 10.3 months		Word	fMRI			
Prisciandaro et al. (54)	15	87	27.50 ± 8.00	DSM-IV	–	–	24 h		Picture	fMRI			
Volkow et al. (55)	36	44	–	DSM-IV	–	–	0		Video	PET			
Kilts et al. (56)^*^	8	50	–	DSM-IV, QMI	–	–			Picture	fMRI	11-point Likert scales	Amygdala, dorsal cingulate cortex	−0.68
Bonson et al. (57)	11	82	32–39	DIS, DSM-IV	6.4	0.33 mg			Picture	PET	Self-report questions	Amygdala, dorsal cingulate cortex	0.74
Kilts et al. (58)^*^	8	0	–	DSM-IV	–	–	2 days	①	Sound	fMRI	Minnesota craving scale	Right subcallosal cortex	−0.89
												Left anterior insula	−0.74
												Brainstem	−0.71
												Left posterior caudate nucleus	−0.77
Sell et al. (59)	10	100	31.6	–	12.40 years	28.75 mg	<11 days	②	Picture	PET			
Hugh Garavan et al. [Hugh (60)]	24	82	34	DSM-IV	-	-			Video	fMRI			
Totals of sample size-weighted averages	150	72	24.59 ± 3.28		4.21 ± 1.53 years	1.35 ± 0.11 mg							
**Cannabis**													
Zhou et al. (61)	51	100	22.94 ± 2.71	DSM-IV	–	–				fMRI			
Karoly et al. (62)	41	53	18.83	DSM-IV, ICD-10	–	–	12 h		Picture	fMRI			
Charboneau et al. (63)	16	31	23.77 ± 3.90	DSM-IV	15.17 ± 2.80 years	2.21 g	8 h		Picture	fMRI			
Cousijn et al. (64)	31	65	21.30 ± 2.30	CUDIT, FTND, MCQ	2.50 ± 1.90 years	5.00 ± 1.50 g			Picture	fMRI			
Ray et al. (65)	10	50	–		–	–	24 h	④	Picture	fMRI			
Filbey et al. (66)	38	81	23.74 ± 7.25	SCID	7.00 ± 7.00 years	3.00 ± 2.00 g	3 days		Item (pipe or pencil)	fMRI			
Totals or sample size-weighted averages	187	71	21.39 ± 3.2		2.56 ± 2.01 years	1.53 ± 0.69 g							
**Methamphetamine**													
Guterstam et al. (67)	40	100	40.1 ± 10.2	DSM-IV	12.60 ± 7.90 years	–	5.2 ± 4.6 days		Video	fMRI			
Grodin et al. (68)	15	80	36.6 ± 8.82	DSM-IV	–	–	9.58 ± 6.58 days	③	Picture	fMRI			
Totals or sample size-weighted averages	55	95	39.29 ± 9.88		12.60 ± 7.90 years	–							
**Polydrug**													
Ray et al. (65)	10	50	–		–	–			Picture	fMRI			
**Tobacco**													
Bi et al. (69)	33	100	19.62 ± 1.89	DSM-V	4.20 ± 1.88 years	15.58 ± 5.53	0		Picture	fMRI	QSU-Brief	Left anterior insula	−0.508
												Right anterior insula	−0.5742
												Left ventromedial prefrontal cortex	−0.494
Zhao (70)^*^	26	100	–	DSM-V	–	–	9 ~ 13 h		Picture	fMRI	QSU-Brief; VAS scale	Right anterior cingulate	0.593
												Right insula	0.432
												Orbitofrontal lobe (*p* = 0.006)	0.533
												Orbitofrontal lobe (*p* = 0.002)	0.585
												Right superior frontal gyrus	0.549
												Right auxiliary motor cortex	0.604
Yang (71)^*^	32	100	26.68 ± 6.28	FTND	8.11 ± 7.02 years	14.41 ± 4.36	0		Picture	fMRI	VAS scale	The PPI between the lDLPFC and the rPHG	0.522
Kathy et al. (72)^*^	78	60	22.57 ± 1.2	FTND	37.53 ± 33.31 months	8.09 ± 1.51	24 h		Video	fMRI	UTS scale	Dorsolateral prefrontal cortex	0.36
												Nucleus accumbens	0.44
Ko et al. (73)	16	100	25.38 ± 3.36	DCIA, DSM-IV-TR	–	–			Picture	fMRI			
Wilson (74)	60	100	33.6 ± 8.5		–	20.90 ± 6.00			Picture	fMRI			
Wilson (74)	82	85	33.0 ± 8.3		–	20.50 ± 5.60	0		Picture	fMRI			
Hartwell (75)	32	44	33.5 ± 11.5	FTND	–	17.70 ± 6.90			Picture	fMRI			
Goudriaan et al. (76)	18	100	35.3 ± 9.4	DSM-IV	–	17.20 ± 3.80			Picture	fMRI			
Weinstein et al. (77)	11	0	45 ± 17	DSM-IV	23.00 ± 13.50 months	26.00 ± 10.00			Video	fMRI			
McClernon et al. (78)	18	39	28.6 ± 7.5	-	11.60 ± 6.70 years	17.80 ± 2.80	–		Picture	fMRI			
McBride et al. (79)	20	50	–	FTND	–	22.00 ± 6.00			Video	fMRI			
Totals or sample size-weighted averages	450	78	25.26 ± 5.95		19.85 ± 14.06 months	15.28 ± 4.31 g							
**Alcohol**													
Bach et al. (80)	115	72	45.6 ± 9.78	DSM-IV	–	–			Picture	fMRI			
Ray et al. (81)	10	70	–	NIAAA	–	6.90 ± 1.90 drinks			Video	fMRI			
Kreusch (82)	12	100	21.30 ± 2.10	AUDIT	–	–			Picture	fMRI			
Courtney (81)	20	70	29.40 ± 9.01	DSM-IV	–	6.42 ± 2.24 drinks			Taste	fMRI			
Vollstädt-Klein (83)	38	0	46.00 ± 9.00	DSM-IV	14.00 ± 10.00 years	120.00 ± 129.00 g	9 ± 5 years		Picture	fMRI			
Vollstädt-Klein et al. (84)^*^	21	57	49.00 ± 11.00	ICD-10, DSM-IV	–	5.00 ± 1.50 drinks			Picture	fMRI	VAS scale	Mesolimbic system	0.32
Ray et al. (65)	10	50	–	Michigan alcohol screening test, alcohol abuse category of the alcohol dependence scale	–	–	24 h		Picture	fMRI			
Park et al. (85)	9	89	23.22 ± 2.48		–	9.16 ± 2.50 drinks			Picture	fMRI			
Myrick et al. (86)	10	80	33.60 ± 11.50	DSM-IV	–	8.17 ± 4.14 drinks	24 h		Picture	fMRI			
Totals or sample size-weighted averages	250	60	36.88 ± 8.1		14 ± 10 years	1.94 ± 0.69 drinks							

* *represents included literature; QSU-Brief is “Brief Questionnaire of Smoking Urges;” UTS Scale is “the Urge to Smoke.” In the “comorbidities” column, “①” means “One met the criteria for nicotine dependence and one met the criteria for marijuana abuse;” “②” means “two used illicit methadone;” “③” means “Marijuana can be positive;” “④” means “Marijuana can be positive;” each blank space indicates that there are no comorbidities or the presence of comorbidities is not mentioned in the literature*.

### ALE Meta-Analysis

#### Single Meta-Analysis Results

There were 32 experiments, 687 subjects, 18 activity enhancement points, and 13 activation clusters with enhanced activity for drug data. The brain regions of drug-dependent patients with enhanced activity after induction by cues were concentrated in the amygdala, hippocampus, middle occipital gyrus, middle temporal gyrus, fusiform gyrus, cingulate gyrus, anterior central gyrus, caudate, middle frontal gyrus, thalamus, and inferior frontal gyrus.

The ALE meta-analysis on alcohol and tobacco included 21 experiments, 687 subjects, 14 activity enhancement points, and 10 activation clusters with enhanced activity. The brain regions of alcohol-dependent patients and tobacco-dependent patients with enhanced activity induced by cues were gathered in the caudate, posterior cingulate gyrus, anterior cingulate gyrus, middle frontal gyrus, thalamus, insula, superior temporal gyrus, and precuneus (see Table 2 in the additional file).

**Table 2:** Single meta-analysis results.

**Illegal drug**						**Alcohol and tobacco**					
**Cluster #**	**Volume (mm^**3**^)**	**x**	**y**	**z**	**Label**	**Cluster #**	**Volume (mm^**3**^)**	**x**	**y**	**z**	**Label**
1	2,072	22.9	−5.2	−20.7	Amygdala, parahippocampal gyrus	1	3,000	−4	14	0	Caudate
2	1,680	−47.9	−66.5	−3.8	Middle occipital gyrus, middle temporal gyrus, fusiform gyrus	2	1,704	−3.7	−47	24.1	Posterior cingulate
3	1,456	−22.2	−6.2	−21.5	Parahippocampal gyrus	3	1,264	−4.4	48.7	−7.2	Medial frontal gyrus
4	1,272	−2	−37.6	28.4	Cingulate gyrus	4	912	−12.3	−14.7	6.6	Thalamus
5	760	47.5	7	26.2	Precentral gyrus	5	904	−4.6	39.8	17.2	Anterior cingulate
6	672	−34.3	−77.4	−24.7	Uvula	6	584	−36.8	11.1	2.2	Insula
7	592	−2.8	16.4	27.7	Cingulate gyrus	7	552	−6	52	−8	Middle frontal gyrus
8	488	7.8	8.9	−10.9	Caudate head	8	408	31.1	−58	48.8	Superior parietal lobule
9	408	−45.6	40.8	14.9	Middle frontal gyrus	9	360	−2.1	−5.2	7.5	Thalamus
10	320	2	−3.2	−15	Hypothalamus	10	256	−28.9	−89.9	10.3	Middle occipital gyrus
11	304	−18.9	−11.2	5.7	Thalamus						

#### Conjunction Meta-Analysis Results

##### Co-activation Area of Nicotine and Drug-Related Data

**Figure 2:**
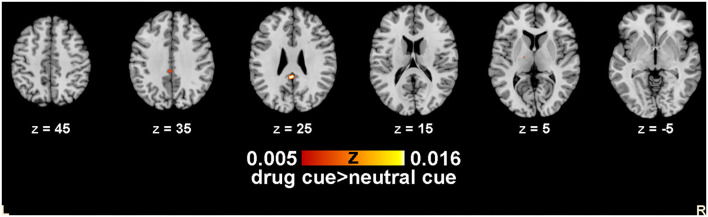
Co-activated clusters about alcohol, nicotine, and illegal drugs. Slices taken at X = 0; Y = −18.

Regarding the comparative ALE meta-analysis of nicotine and drugs, five activity enhancement points and three activation clusters with enhanced activity were generated. The brain areas co-activated by the two were the posterior cingulate and caudate (see Table 3, Figure 2).

**Table 3:** Co-activated clusters about alcohol, nicotine, and illegal drug.

**Cluster #**	**Volume (mm^**3**^)**	**x**	**y**	**z**	**Extrema value**	**Label**
1	472	−1.5	−40.1	28.3	0.020584242	Posterior cingulate
2	32	10	11.5	−8	0.015853202	Caudate
3	16	−16	−12	5.1	0.01629886	Thalamus

### Comprehensive Meta-Analysis Results

#### Heterogeneity Test and Publication Bias Test

First, the Heterogeneity test was performed. The *Q*-test result was significant (*P* < 0.001), indicating that the effect sizes of the original research were not similar.

**Figure 3:**
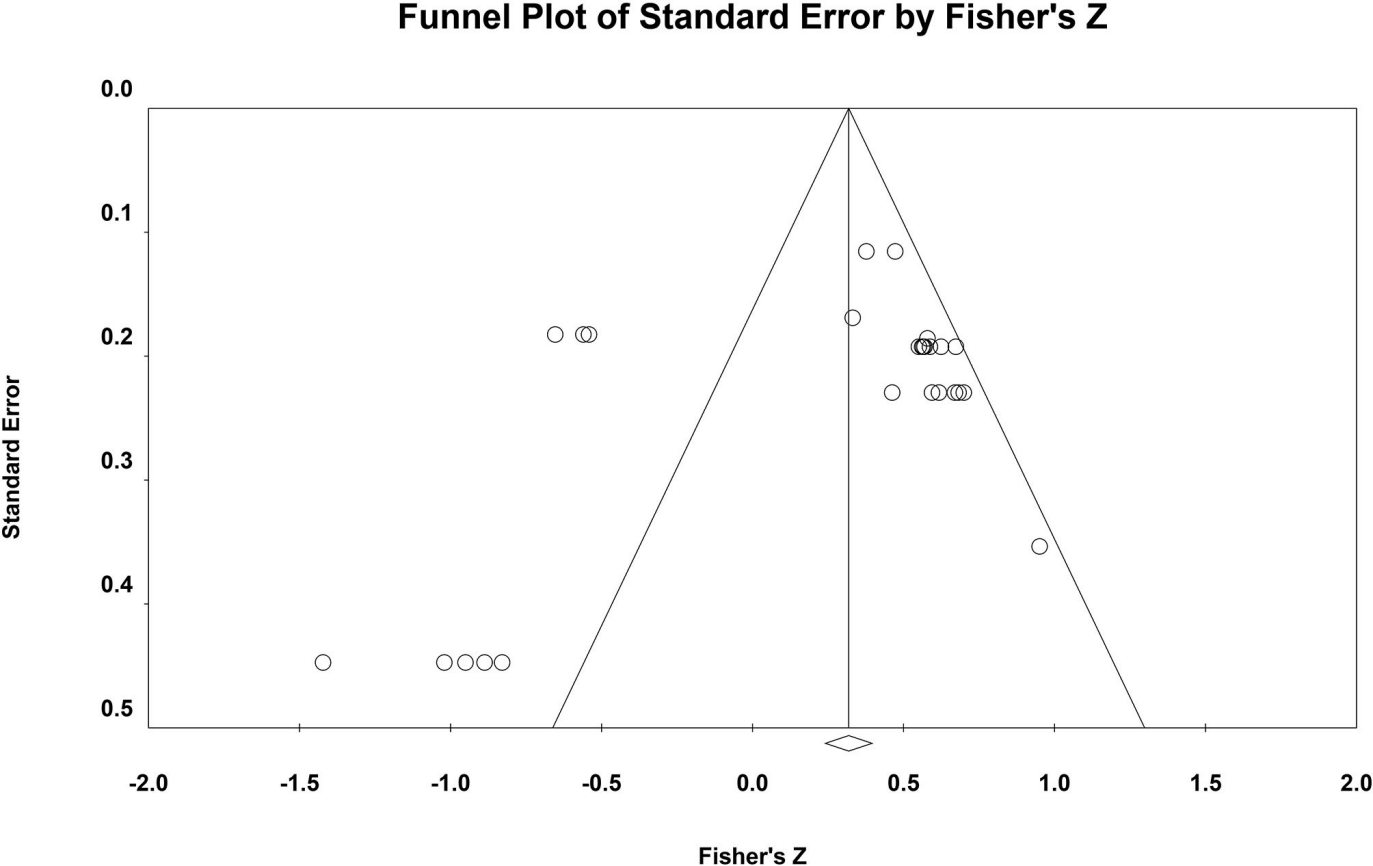
Funnel plot. The vertical axis is the log standard error of the effect size, the horizontal axis is the effect size, the inside of the funnel is the confidence interval, and the central axis is the combined effect size.

Second, the publication bias of this meta-analysis was checked by a funnel plot (see Figure 3).

Regarding the funnel plot, the point on the left is farther from the axis of symmetry than the point on the right. This distribution characteristic indicates the possible occurrence of publication bias. Because the funnel plot is a preliminary check from a subjective point of view, we further performed Rosenthal's Failsafe N and Egger's tests to more accurately test the possibility of publication bias (see Table 4).

**Table 4:** Publish deviation test results.

**Rosenthal's *N***	**Egger's intercept**	**SE**	**LL**	**UL**	***p***
238	1.71	01.40	−5.27	0.49	>0.05

*LL and UL respectively represent the lower and upper limits of Egger's Intercept's 95% confidence interval*.

According to the Egger's test, the results suggest that there is no publication bias. From Rosenthal's *N*-value, it is necessary to include 238 (<2,200) articles to neutralize the two total effect sizes, indicating the presence of publication bias in this study.

Of the three publication bias tests described above, two results (funnel plot and Rosenthal's N) indicated the presence of publication bias and one result (Egger's test) indicated the absence of publication bias, and no results were obtained for all three tests. Therefore, further analysis is still required and the Trim and Fill method needs to be employed to examine the effect of publication bias on the results of the meta-analysis.

The Trim and Fill method proposed by Duval and Tweedie was further used to test the influence of publication bias on the results of meta-analysis (87). It was found that after trimming and filling the research literature, the overall effects obtained by using the random effects model were still significant. In addition, our unpublished literature represents 14.3%, which is already a significant proportion. Taken together, these results suggest that although there may be a slight publication bias in this study, the main findings of the meta-analysis are valid. Thus, although there may be publication bias in the two meta-analyses in this study, the main conclusion drawn from the comprehensive meta-analysis is valid.

#### Main Effect

The relationship between brain imaging data and craving scores was tested from an overall perspective. The results show that there are a total of 26 independent effect sizes, with the total subjects number of 6,663, and the overall correlation coefficient of 0.222 (see Table 5).

**Table 5:** Random effects model analysis results.

***N***	**k**	**r**	**LL**	**UL**	**Z**	***p***
260	26	0.222	0.025	0.402	2.203	<0.05

*N represents sample size, K represents number of studies, and LL and UL respectively represent lower limit and upper limit of 95% confidence interval of R*.

## Discussion

### Co-activated Brain Regions

#### Posterior Cingulate

Findings indicate that the main co-activated brain area of tobacco-, alcohol-, and drug-related data is the posterior cingulate cortex (PCC); its voxel is far more than other co-activated brain areas. The PCC's most common identifier in the addiction field is as the self-function center of the default mode network (DMN), which is mainly responsible for the processing of “self” information such as autobiographical recall, self-evaluation, and reflection of one's own emotional state (88). In general, PCC guides attention to the internal (89), transmitting internal information for further evaluation via the ventromedial prefrontal lobe (mPFC) (90). Previous studies have found that changes in the PCC gyrus of different drug-dependent patients in craving states are often closely related to the DMN (91). In heroin-dependent patients, the PCC → mPFC pathway is activated in the process of reducing the significance of drug-related cues (92). After 24 h abstinence in alcohol-dependent patients, PCC has high synchronicity with other parts of the DMN (93). PCC damage can even lead to the disappear of drug cravings and its damage causes tobacco-dependent patients to lose interest in smoking tobacco (94). Regarding concomitant substance use, attention should be paid to cultivating patients' positive self-concept to enhance withdrawal motivation and mitigate relapses. Simultaneously, attention should be paid to the self-identity of successful abstainers to allow them to fully integrate into social groups and resume normal work and life.

#### Caudate

The caudate is the second co-activation area. Habit formation is a cause of substance addiction and, here, the caudate produces neuronal responses (95). Using reward methods for individuals form conditioned reflexes is an effective way to form habits and the caudate and related cortical-striatal loop brain regions are crucial parts of the addiction reward loop. This suggests that the caudate may promote the formation of drug-taking habits through the activation of reward loops. Additionally, the caudate participates in the cognitive process of inhibiting control (96, 97). The dual disorders of cognitive control and craving processing can cause addiction. The activation of the caudate in drug craving is beneficial for inhibiting relapse behavior; however, it cannot effectively inhibit the spontaneous activities of DMN in heroin-dependent patients, thus it cannot perform cognitive control on some target-directed activities (e.g., seeking drugs, drug use) (98). Therefore, the caudate, a part crucial to the brain's learning and memory, accelerates the addiction process. Its control function allows it to inhibit individual relapse to a certain extent in the craving state, but abnormal changes in the caudate may explain why patients cannot control relapses or take other drugs to relieve their cravings. Treatment providers should pay more attention to cognitive control training for people who use substances concomitantly, such as high-intensity interval training, mindfulness training, and cognitive behavioral therapy.

#### Thalamus

The thalamus is the third co-activation area. As a sensory center, thalamus abnormality can cause patients to disassociate themselves from reality (99). After ketamine enters the human body, it inhibits the thalamus-neocortical system, selectively blocks pain, and activates the limbic system leading to excitement; the combination of alcohol with GABA_A_ receptors in the thalamus makes people unresponsive as they temporarily detach from painful realities (100, 101). Here, the thalamus is also an important part of the memory system and addiction memory often causes relapse (102). The thalamus downstream loop is closely related to addiction-related memory: the PVT → CeA loop is the key neural pathway for the formation of drug addiction memory and is responsible for connecting rewards produced by opioids with the environment; the PVT → NAc → LH loop is important for maintaining addiction-related memory. Through optogenetic and other technical means, the PVT → NAc or NAc → LH pathway can be manipulated in the memory extraction stage to eliminate addiction-related memory, for preventing relapse (103). It can be seen that the thalamus is like an eraser that erases the memory of addiction. The two subregions of the thalamus are also involved in cognitive control and craving, revealing the implications of the thalamic subnucleus in the pathology of acute abstinent heroin users (104). Thus, the thalamus has become a new focus for solving drug addiction. Regarding concomitant substance abuse, the “eraser” is a new development proposed for wiping addiction-related memory from patients during detoxification.

### Therapeutic Implications From Three Overlaps

Tobacco, alcohol, and drug-dependent patients will process self-information in a craving state. Relevant studies have shown that self-concept is related to drug craving (105). Drug users adopt negative coping mechanisms when facing social pressure or pressure caused by drug withdrawal because of their low self-concept (106). Additionally, self-concept is positively correlated with the motivation of drug withdrawal (107, 108), which is an important factor in the treatment of craving (109). Notably, the self-concept of drug use involves a drug-use identity the degree to which drug use behavior is included in the self-concept by the drug-dependent patients. The higher the level of inclusion, the higher the identity of drug use. Drug-use identity can significantly predict drug craving, as confirmed in alcohol, tobacco, and drug use (110–114). Furthermore, substance users' drug craving has a cross-cue response mode when they try to withdraw from one addictive substance, and continuous exposure to another drug may induce craving for both substances, thus increasing the possibility of treatment failure (115). Therefore, drug-dependent patients may also experience drug cravings under tobacco and alcohol cues, arousing drug-use identity and resulting in a low sense of self-identity and loss of determination to abstain from drug-use.

That said, addiction-related memory (a pathological memory formed by repeatedly associating the pleasure of drugs with the drug-use environment) is activated by patients' craving state. Like other long-term memories, addiction-related memory contains both narrative scenarios and emotional memories such as reward memories, habitual actions, and drug-use techniques that are formed during long-term drug use and belong to procedural memory (116). Therefore, tobacco and alcohol-dependent patients may activate the reward circuit in the craving state, producing conditioned reflexes and abnormal reward circuits that may cause drug abstainers to relapse (117).

Thus, both self-information processing and the arousal of addiction-related memories can trigger relapses. However, in the current social status of addiction treatment, many people mistakenly think that focusing on drug rehabilitation and ignoring tobacco and alcohol withdrawal or using them to replace drugs are effective treatments. In fact, such treatments may cause drug-dependent patients with tobacco and alcohol addiction to give up on themselves because their identity of drug use is induced by craving for tobacco and alcohol after successful drug withdrawal, and they may regard themselves as patients in their mind. At the same time, the reward memory in the addiction-related memory will induce conditioned reflexes and activate the action of drug use. Therefore, drug-dependent patients can start to abstain from tobacco and alcohol in the early stage of detoxification, so as to avoid the tragedy of “penny wise and pound foolish” at a later stage.

### Relationship Between Brain Imaging and Subjective Craving

We found that only nine of the 51 studies reported a correlation between craving scores and activated brain regions. Therefore, this result (*r* = 0.222) does not fully indicate that ALE meta-analysis results can be represented by craving but it suggests to some extent that the accuracy of neuroimaging indirect measurement of craving needs to be improved. Neuroimaging provides a quantitative measurement for the evaluation of drug craving. However, these results can only show that neurophysiological changes are related to craving, and they cannot prove that there is a causal relationship between these factors. Sayette et al. (118) proposes that craving and hunger are both subjective experiences of the desire to ingest a substance, they are not necessarily related to physiological signals, and neither is necessarily related to physiological indicators that express biological needs. However, (for example, the blood sugar level in the circulation when hunger does not necessarily decrease), but both can be stimulated by environmental stimuli (such as stimulated by signals that indicate availability). A study also shows that craving and relapsing do not depend on direct physiological drug effects (119). Furthermore, the ecological validity of the cue-induced paradigm is poor, as the subject may be affected by response tendency and social expectations, which may influence the correlation between brain activation and craving scores.

## Limitations and Future Implications

There are few published studies on cravings for new drugs, and the proportion of new drugs explored in this study is low, thus further work is needed to improve the representativeness of the current status of drug dependence. Conditions that induce craving are mostly shown in pictures, so the retrieved literature is not enough to conduct a comparative meta-analysis of brain activation induced by different cues.

Future research can examine related unpublished research on new drugs, emerging conditions for induction, and different imaging conditions to supplement the literature and correct the unpublished deviations of meta-analysis. Concerning craving in drug addiction, researchers should consider current social situations and increase research efforts on new drug addiction in future studies. Additionally, scholars should actively explore experimental conditions that can better induce real psychological craving, such as the use of multi-sensory stimulation, and specific conditions for induction should be formulated based on different regions and drugs.

## Conclusion

The co-activation areas of tobacco, alcohol, and drug-dependent patients induced by cues are mainly the PCC, followed by the caudate and thalamus. The PCC is closely related to the DMN and is the main component of the DMN self-function center; the caudate and thalamus are both related to addiction-related memory. This indicates that the three drug cravings all involve the processing of self-information and the initiation of addiction-related memories.

Because these cravings induce the processing of self-information, including self-concept, drug-dependent patients will stimulate their drug-use identity. As these drug abstainers may induce drug cravings under tobacco and alcohol cues, they may also arouse drug-use identity under these cues, thereby increasing the rate of relapse. Moreover, addiction-related memories evoked under tobacco and alcohol cues include reward memories, which can activate drug abstainers' reward circuits, produce a conditioned reflex, and cause relapse. Therefore, professionals should pay attention to tobacco and alcohol withdrawal in the early stage of drug rehabilitation.

This study found that neuroimaging only mildly represents subjective craving. Thus, researchers should not use neuroimaging results exclusively to represent subjective craving. Furthermore, the ecological validity of the environment for cue-induced craving should be increased in the laboratory to improve the present research.

## Author Contributions

HL: study concept and design. YL, JX, YQ, and QZ: literature acquisition. HH: data analysis and interpretation. DZ: manuscript preparation and editing. YL: guidance of methodology and language revision. YJ: data curation and editing of writing. All authors contributed substantially and according to Frontiers in Psychiatry guidelines to be recognized as authors. All authors have read and approved the final version of the manuscript.

## Funding

This study was supported by the National Social Science Foundation of China (Grant No. 20BSH047).

## Conflict of Interest

The authors declare that the research was conducted in the absence of any commercial or financial relationships that could be construed as a potential conflict of interest.

## Publisher's Note

All claims expressed in this article are solely those of the authors and do not necessarily represent those of their affiliated organizations, or those of the publisher, the editors and the reviewers. Any product that may be evaluated in this article, or claim that may be made by its manufacturer, is not guaranteed or endorsed by the publisher.
